# Exploring the Relationship Between Protein-Level Ratios (rQLTs) and Duodenal Ulcer

**DOI:** 10.3390/cimb48060643

**Published:** 2026-06-22

**Authors:** Siwen Tang, Yongwei Li, Xi Yu, Ying Xiao, Tian Zhong

**Affiliations:** Faculty of Medicine, Macau University of Science and Technology, Taipa, Macao 999078, China; 3230004447@student.must.edu.mo (S.T.);

**Keywords:** duodenal ulcers (DU), Mendelian randomization (MR), protein-level ratios (rQTLs)

## Abstract

To explore the associations between protein-level ratios (rQLTs) and duodenal ulcer (DU) risk using Mendelian randomization (MR), colocalization, and pathway analysis approaches. A bidirectional MR approach was used to identify molecular targets linking rQLTs with DU, employing the inverse variance weighted (IVW) method for causal estimation. Colocalization analysis ensured the reliability of inferred causal relationships. Gene interaction networks were constructed via STRING, and key regulatory hub-genes were identified through Cytoscape analysis. Significant inverse associations were found between *rQLT-ACE2/GGT1* (Angiotensin-converting enzyme 2/γ-glutamyl transpeptidase 1) (IVW, OR (95% CI) = 0.754 (0.674–0.843), adjusted *P*_IVW_ = 0.0005), and DU risk in the East Asian (Japanese) population. No statistically significant associations were observed in the European population. The findings indicate a genetic inverse association between *rQLT-ACE2/GGT1* and DU risk in the East Asian (Japanese) population, while no corresponding association was observed in Europeans. These results provide genetic evidence consistent with a potential association rather than causal inference or biomarker validation. This study does not support conclusions regarding diagnostic or therapeutic utility at this stage.

## 1. Introduction

Duodenal ulcer (DU) is a chronic peptic ulcer predominantly affecting the duodenal bulb. In Western populations, DU represents the most prevalent form of peptic ulcer disease, with population-based studies reporting a lifetime prevalence of 5–15%. In contrast, epidemiological surveys in China estimate a prevalence of around 10% [1].

The pathogenesis of DU is not only associated with inflammatory responses [2] but also closely linked to dysbiosis of the intestinal microbiota [3]. These pathogenic factors are accompanied by coordinated alterations in circulating protein expression profiles involved in immune and metabolic processes.

In this study, the exposure consists of protein-level ratio traits (rQLTs), defined as quantitative traits representing the ratio of circulating plasma levels between two proteins (protein A/protein B). A total of 2821 rQLTs were included as exposures in the analysis. These ratio traits capture relative protein abundance rather than absolute protein levels and reflect the biological balance between functionally related proteins within systemic pathways [4].

Accordingly, rQLTs can be interpreted as continuous quantitative variables, where higher or lower values indicate a shift in the relative abundance between two proteins. This framework enables the investigation of genetically influenced variation in protein homeostasis and its relationship with disease risk.

Therefore, this study systematically investigates the associations between 2821 genetically predicted rQLTs and DU risk using Mendelian randomization (MR), colocalization, and pathway analysis approaches. The objective is to evaluate genetic evidence for potential associations between circulating protein ratio traits and DU risk, rather than to identify biomarkers or infer clinical utility.

## 2. Materials and Methods

Statistical analyses were performed using R software (version 4.0.2, Posit Software, PBC, Boston, MA, USA). Network visualization and analysis were conducted using Cytoscape software (version 3.10.3, Institute for Systems Biology, Seattle, WA, USA).

### 2.1. Data

We obtained the genotype data from the genome-wide association studies (GWAS).

#### 2.1.1. Exposure Data

Genetic variants for rQLTs were obtained from publicly available GWAS summary statistics (https://www.ebi.ac.uk/gwas/, accessed on 30 December 2025), accession numbers GCST90313126–GCST90315946. The original study was based on Olink proteomics data from more than 54,000 UK Biobank participants with circulating protein measurements for 1463 proteins.

After quality control and restriction to European ancestry individuals, a total of 43,509 participants were included in the final analysis, from which 2821 rQLTs were derived, representing 4248 significant protein–protein ratio associations.

#### 2.1.2. Outcome Data

Genetic variants for DU in East Asian (Japanese) populations were obtained from the IEU OpenGWAS database (https://www.ebi.ac.uk/gwas/, accessed on 30 December 2025), accession number GCST90270928. This dataset includes 252,639 individuals, comprising 11,964 cases and 240,675 controls. The DU phenotype was defined based on clinically diagnosed duodenal ulcer cases derived from electronic health records, primarily using ICD-10 codes (K25 and K26 series), with additional harmonized phenotype mappings including PheCode 531.3. These definitions were constructed from hospital records and integrated clinical coding systems to identify DU-related cases in large-scale biobank data.

Genetic variants for duodenal ulcer in European (UK) populations were obtained from the IEU OpenGWAS database (https://www.ebi.ac.uk/gwas/, accessed on 30 December 2025), accession number GCST90044132. This dataset includes 456,348 individuals, comprising 1484 cases and 454,864 controls. The reported trait was defined using PheCode 531.3, which corresponds to duodenal ulcer phenotypes derived from electronic health record-based ICD coding (ICD-10 K26 series) within the UK Biobank system.

All summary statistics were accessed from the IEU OpenGWAS database on 30 December 2025. The original GWAS publications did not report overlapping samples between the exposure and outcome datasets used in this Mendelian randomization analysis; therefore, potential overlap is considered unlikely.

### 2.2. Methods

#### 2.2.1. Mendelian Randomization (MR) Analysis

##### Forward MR Analysis

**Figure 1:**
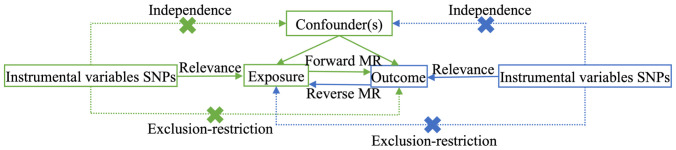
Flowchart of the MR design.

Forward MR analysis was conducted based on the three assumptions (Figure 1). To ensure the validity and reliability of instrumental variables (IVs), a multi-step selection procedure was applied. First, genetic variants associated with rQLTs were selected using a significance threshold of *p* < 1 × 10^−5^. This threshold was adopted instead of the conventional genome-wide significance level (*p* < 5 × 10^−8^) because protein ratio quantitative traits typically yield fewer genome-wide significant loci due to their derived nature and reduced statistical power compared with single-protein traits. Therefore, a relaxed but commonly used threshold in proteomic and protein-ratio MR studies was applied to ensure sufficient instrumental variables for downstream causal inference. Second, linkage disequilibrium (LD) pruning was performed using an r^2^ threshold of 0.001 within a 10,000 kb window to ensure independence among single nucleotide polymorphisms (SNPs). Third, instrumental strength was evaluated using the F-statistic (F = β^2^/SE^2^), and SNPs with F < 10 were excluded to minimize weak instrument bias; summary F-statistics of the retained instruments were calculated for quality control. Fourth, allele harmonization was performed to ensure consistency of effect alleles between exposure and outcome datasets, with palindromic SNPs resolved using allele frequency information when available or otherwise excluded. The number of SNPs removed at each stage, including LD pruning and harmonization, was systematically recorded and reported in the corresponding results tables.

**Figure 2:**
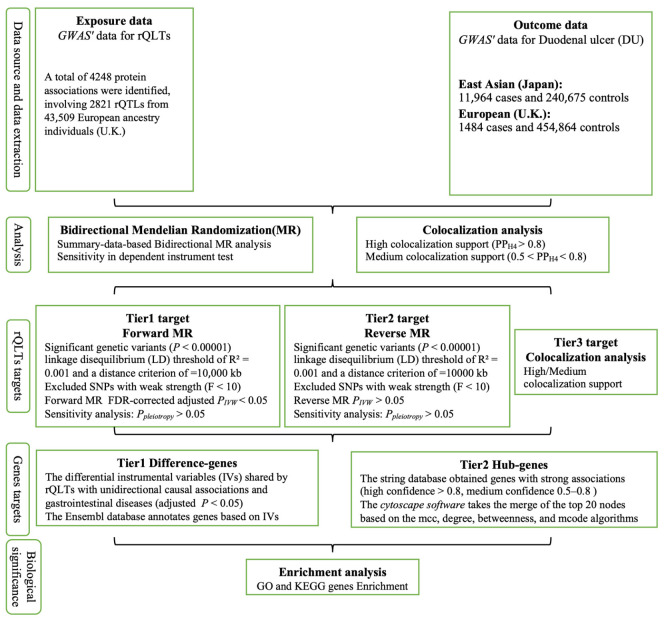
Flowchart of the study design.

Assessment of horizontal pleiotropy and heterogeneity was conducted using multiple complementary approaches. Directional pleiotropy was evaluated using the MR-Egger regression intercept test, while heterogeneity among SNP-specific estimates was assessed using Cochran’s Q statistic. In addition, the MR-PRESSO framework was applied, including global test for horizontal pleiotropy, outlier detection, and correction analysis after removal of identified outlier SNPs. When outliers were detected, causal estimates were recalculated after outlier exclusion, and both original and corrected results were compared. The inverse variance weighted (IVW) method was used as the primary MR estimator under the assumption of no horizontal pleiotropy. Multiple testing correction was performed using the Benjamini–Hochberg false discovery rate (FDR), with statistical significance defined as adjusted *p* < 0.05. All analyses were conducted using R software (version 4.4.0) [5] (Figure 2).

##### Reverse MR Analysis

Reverse MR analysis was performed to assess the potential causal effect of DU on rQLTs. In this analysis, DU was treated as the exposure and rQLTs as outcomes. Genetic instruments for DU were obtained from published GWAS summary statistics.

Instrument selection, LD clumping, instrumental strength assessment, and allele harmonization were conducted using the same procedures and parameters as described in Section Forward MR Analysis.

#### 2.2.2. Colocalization Analysis

A Bayesian colocalization analysis was conducted using the coloc R package to evaluate whether rQLTs and gastrointestinal disease traits share a common causal variant within the same genomic region [5]. For each locus, a predefined genomic window of ±250 kb around the lead SNP was used to extract summary statistics from both exposure and outcome datasets. Only SNPs present in both datasets were included, and the number of overlapping SNPs at each locus was recorded to ensure transparency of the colocalization input data.

The analysis was performed under the standard coloc framework assuming a single causal variant per trait within each locus. No conditional analysis was performed prior to colocalization. Default prior probabilities were applied, with p1 = 1 × 10^−4^ (probability that an SNP is associated with trait 1), p2 = 1 × 10^−4^ (probability that an SNP is associated with trait 2), and p12 = 1 × 10^−5^ (probability that an SNP is associated with both traits). The posterior probability of hypothesis 4 (PP_H4_) was used as the primary metric to assess evidence for colocalization. A PP_H4_ value > 0.8 was considered strong evidence supporting a shared causal variant, while values between 0.5 and 0.8 were interpreted as suggestive evidence.

#### 2.2.3. Identification and Functional Enrichment Analysis of Hub-Genes

##### Identification and Screening of Hub-Genes Targets

(a) The identification of hub genes was performed through a multi-step integrative workflow to ensure reproducibility. First, single nucleotide polymorphisms (SNPs) showing significant discrepancies between exposure and outcome associations (adjusted *p* < 0.05) were selected. Duplicate SNPs were removed by retaining the variant with the lowest *p*-value. These SNPs were then mapped to genes using the Ensembl database based on genomic location (positional mapping within ±10 kb of gene boundaries), and SNP-level signals were aggregated at the gene level to generate an initial candidate gene list.
(b) Protein–protein interaction (PPI) analysis was conducted using the STRING database (https://cn.string-db.org accessed on 30 December 2025) with a high-confidence interaction threshold (combined score > 0.7). The resulting interaction network was imported into Cytoscape software for downstream network analysis. Hub gene identification was performed using the CytoHubba plugin based on three centrality algorithms: maximal clique centrality (MCC), degree, and betweenness centrality. In parallel, module detection was performed using the MCODE plugin with the following parameters: node score cutoff = 0.2, k-core = 2, degree cutoff = 2, and haircut option enabled.
(c) For hub gene selection, genes ranked within the top 20 nodes in each centrality algorithm (MCC, degree, and betweenness) were first identified. The final hub genes were defined as those genes that (i) consistently ranked within the top 20 across all three centrality measures and (ii) were also included in the highest-scoring MCODE module. The overlap between these selection criteria hub genes.

##### Enrichment Analysis of Hub-Genes

GO and KEGG enrichment analyses were conducted on the hub-genes to determine the top 10 significantly enriched pathways within each category—biological process (BP), cellular component (CC), molecular function (MF), and Kyoto Encyclopedia of Genes and Genomes (KEGG) pathways—using an adjusted *p* < 0.05. The results demonstrated that at least one biologically relevant functional pathway was significantly enriched.

## 3. Results

### 3.1. The Causal Association Between the rQLTs and DU

**Figure 3:**
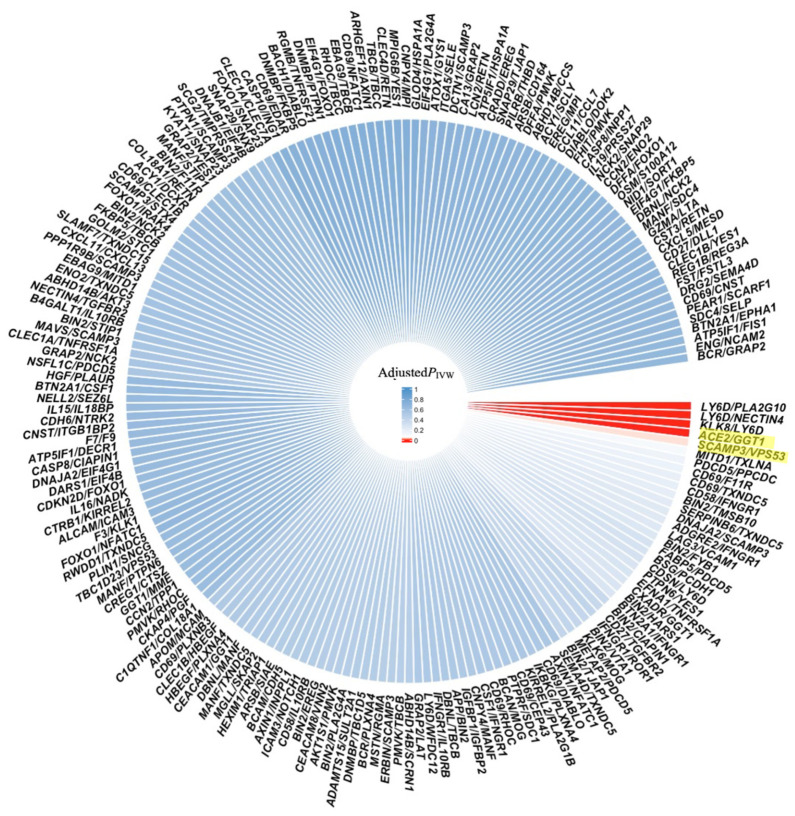
Causal relationships among rQTLs associated with DU in East Asian were examined. Of the 2821 rQTLs analyzed, only 2 showed a unidirectional causal association with DU in this population (Reverse MR, *P*_IVW_ < 0.05). In the network visualization, red edges indicate statistically significant causal relationships, whereas blue edges represent non-significant associations in the forward MR analysis, the yellow-highlighted rQTLs was identified as having a reliable unidirectional causal association after validation through bidirectional MR analysis.

**Figure 4:**
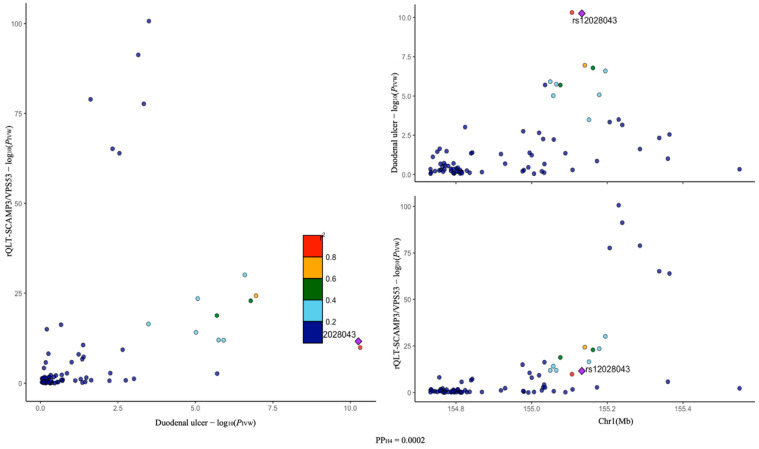
*rQLT-SCAMP3/VPS53* demonstrates colocalization with DU in East Asian.

East Asian (Japanese): Forward MR analysis identified 5 rQLTs (*rQLT-ACE2/GGT1*, *rQLT-KLK8/LY6D* (Kallikrein-8/Lymphocyte antigen 6 family member D), *rQLT-LY6D/NECTIN4* (Lymphocyte antigen 6 family member D/Nectin cell adhesion molecule 4), *rQLT-LY6D/PLA2G10* (Lymphocyte antigen 6 family member D/Group 10 secretory phospholipase A2), *rQLT-SCAMP3/VPS53* (Secretory carrier-associated membrane protein 3/Vacuolar protein sorting-associated protein 53)) as significantly associated with DU among East Asian (FDR-adjusted *P*_IVW_ < 0.05). Reverse MR analysis revealed evidence of reverse causality for 3 rQLTs (*rQLT-KLK8/LY6D*, *rQLT-LY6D/NECTIN4*, *rQLT-LY6D/PLA2G10*). As a result, only 2 rQLTs (*rQLT-SCAMP3/VPS53 and rQLT-ACE2/GGT1*) were retained as potentially exerting causal effects on DU in East Asian (Figure 3). Importantly, colocalization analysis of *rQLT-SCAMP3/VPS53* and DU (Japanese) showed no shared causal variant, suggesting limited stability of the inferred causal relationship (PP_H4_ = 0.0002) (Figure 4). Therefore, the findings provide robust evidence that changes in the *rQLT-ACE2/GGT1* exert a causal influence on DU risk among East Asian (Japanese) (Table 1).

**Figure 5:**
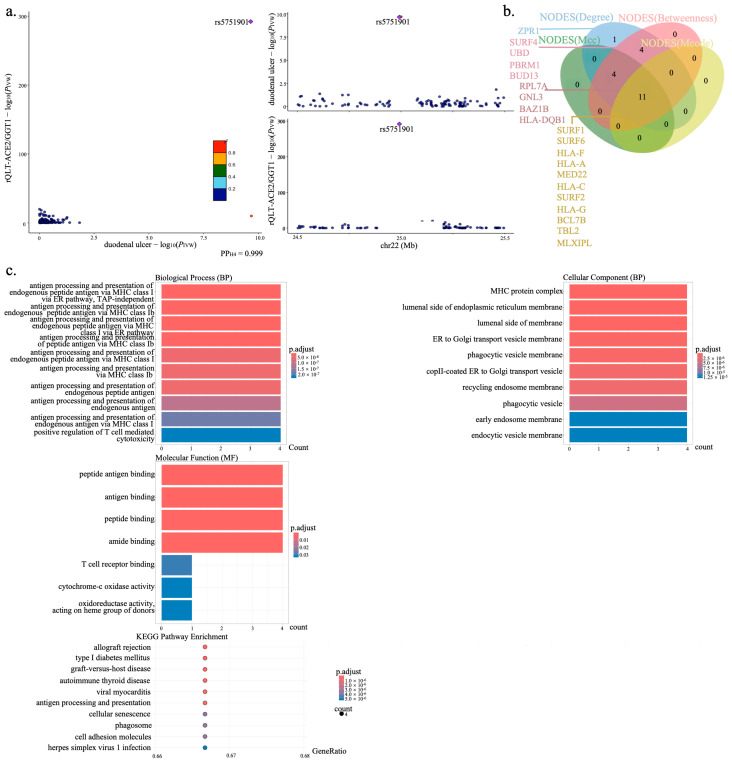
(**a**) *rQLT-ACE2/GGT1* demonstrates colocalization with DU in East Asian. (**b**) The Venn diagram illustrates the overlap between *rQLT-ACE2/GGT1*-associated genes and confirmed genetic loci for DU in East Asian, with the merged region representing hub-genes. (**c**) Functional enrichment analyses, including GO and KEGG pathway analysis, were conducted on the hub-genes.

**Figure 6:**
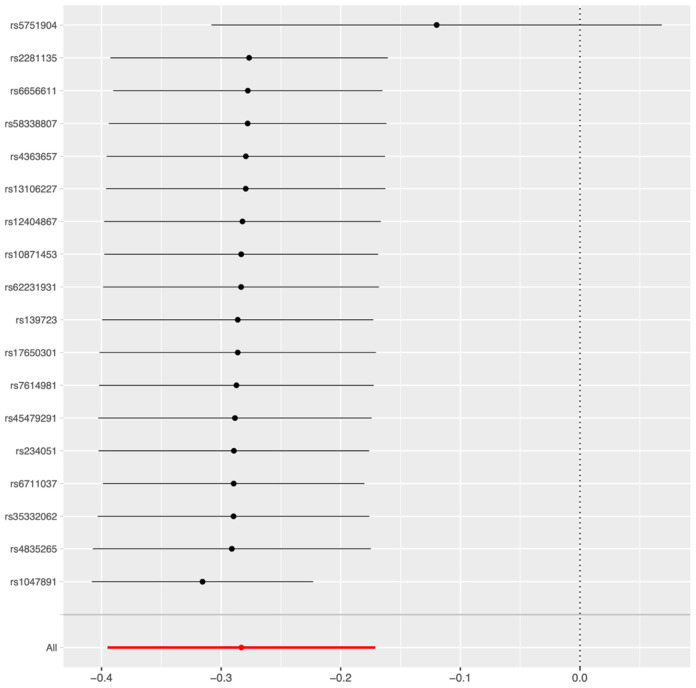
MR leave-one-out sensitivity analysis for ‘*rQLT-ACE2/GGT1*’ on ‘DU (Japanese)’. Each point represents the causal effect estimate after omitting a single individual SNP. All confidence intervals lie on the same side of the null dashed line, indicating that the causal estimate is statistically stable and not driven by any single genetic variant.

*rQLT-ACE2/GGT1* demonstrates protective effects against DU in Japan (IVW, OR (95% CI) = 0.754 (0.674–0.843), Adjusted *P*_IVW_ = 0.0005). The association shows no evidence of heterogeneity or horizontal pleiotropy (*P*_Heterogeneity_ = 0.077, *P*_Pleiotropy_ = 0.216), and reverse causality was not detected in bidirectional MR analysis (*P*_IVW_ = 0.592). Colocalization analysis provides support for the presence of a shared causal variant (rs5751901) and indicates a highly robust causal relationship (PP_H4_ = 0.999) (Table 1, Figure 5a). Moreover, leave-one-out sensitivity analysis confirms the stability and robustness of the observed association (Figure 6).

**Figure 7:**
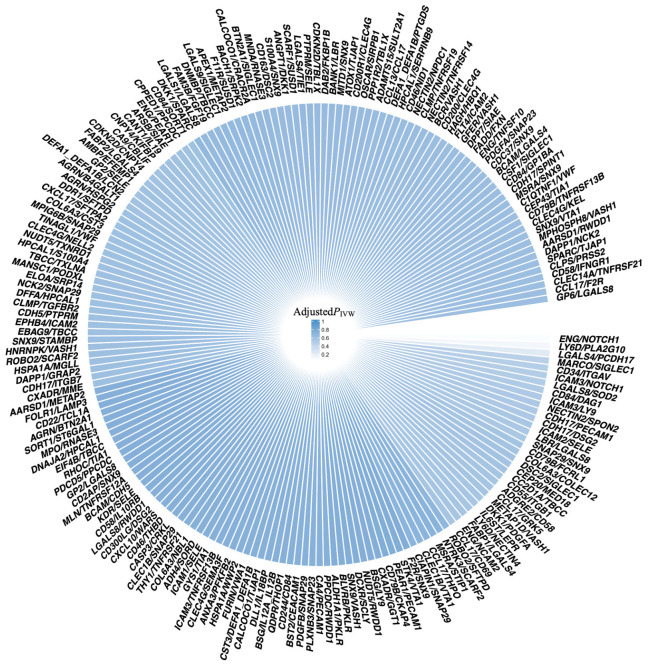
Causal relationships among rQTLs associated with DU in European were examined. Of the 2821 rQTLs analyzed, 0 showed a unidirectional causal association with DU in this population (Reverse MR, *P*_IVW_ > 0.05).

European (UK): MR analysis revealed no statistically significant associations between any of the 2821 rQLTs and DU risk in European (FDR-adjusted *P*_IVW_ > 0.05) (Figure 7).

### 3.2. Functional Enrichment of Hub Genes in the rQLT-ACE2/GGT1–DU (Japanese) Network

The *rQLT-ACE2/GGT1*-associated DU (Japanese) network comprised 11 hub genes (*SURF1*, *SURF6*, *HLA-F*, *HLA-A*, *MED22*, *HLA-C*, *SURF2*, *HLA-G*, *BCL7B*, *TBL2*, and *MLXIPL*) (Table 2, Figure 5b). GO and KEGG pathway enrichment analysis of this gene set identified the top 10 significantly enriched biological processes (Figure 5c). The enriched pathways were predominantly immune-related and included antigen processing and presentation, allograft rejection, and viral myocarditis-related pathways.

## 4. Discussion

This study investigated the association between rQLTs levels and DU in both Japan and U.K. We systematically analyzed the molecular mechanisms of disease-related target genes within the biological pathways implicated in these conditions. The integration of GO and KEGG enrichment analyses revealed that the *rQLT-ACE2/GGT1*–associated gene set is primarily enriched in immune-related biological pathways rather than indicating specific disease mechanisms.

### The Protective Effect of rQLT-ACE2/GGT1 Levels in DU (Japanese)

*ACE2* is a membrane-bound enzyme that is highly expressed in the intestine, where it serves as a key marker for brush border proteins and differentiated intestinal epithelial cells. It plays an essential role in maintaining both the structural and functional integrity of the intestinal barrier [6,7]. With its well-documented anti-inflammatory, antioxidant, and anti-fibrotic properties [8], *ACE2* reduces tissue damage, regulates cell proliferation and migration, and promotes the regeneration of intestinal epithelial cells [7]. However, the current study does not directly test these functional effects, and such interpretations are based on prior literature rather than enrichment output.

In contrast to *ACE2*, *GGT1* predominantly participates in glutathione metabolism and the regulation of cellular antioxidant defenses. Glutathione is a key endogenous antioxidant, and fluctuations in its levels significantly affect cellular susceptibility to oxidative stress. Excessive oxidative stress can lead to cell injury and apoptosis, which in turn compromise tissue function [9]. *GGT1* has been implicated in the regulation of redox balance; however, the present study does not directly assess oxidative stress pathways, and such interpretations are based on prior biological knowledge rather than enrichment results. Furthermore, accumulating evidence suggests that *GGT1* activity may be associated with inflammatory signaling pathways, including *NF-κB* activation [10]. Nevertheless, this association should be interpreted cautiously, as it is not directly supported by the enrichment analysis performed in this study. While oxidative stress–related and inflammatory processes are biologically plausible in gastrointestinal injury, they were not specifically identified among the top enriched pathways in the current dataset. Collectively, *ACE2* and *GGT1* may reflect distinct biological roles in intestinal physiology, but the current analysis does not provide evidence for functional antagonism, directional effects, or causal relationships in DU pathogenesis. Their potential involvement should therefore be considered exploratory and hypothesis-generating.

This study identifies a genetic association between *rQLT-ACE2/GGT1* and DU risk in Japanese, based on MR and colocalization analyses, without implying therapeutic effects. GO and KEGG enrichment analyses of hub-genes suggest that the associated gene set is enriched in immune-related biological pathways. First, all nucleated cells are capable of expressing *MHC-I* molecules. *rQLT-ACE2/GGT1* regulates antigen processing and presentation pathways by enhancing both classical *MHC-I* and non-classical *MHC-Ib* signaling, thereby modulating immune responses [11]. *MHC-I*-mediated antigen presentation is indispensable for *CD8^+^ T* cell activation. Upregulated *MHC-I* expression enhances the capacity of *CD8^+^ T* cells to recognize and eliminate invading pathogens, thereby reinforcing immune surveillance, counteracting immune evasion, and playing a central role in adaptive immunity [12,13]. These pathway annotations suggest potential involvement in immune regulation; however, no functional effects in DU are directly demonstrated in this study. Second, Helicobacter pylori (HP) is a primary etiological agent in DU pathogenesis and is prone to aggregated colonization in specific populations [14]. The enrichment of immune-related pathways, including those associated with graft-versus-host disease, reflects general immune system involvement rather than disease-specific mechanisms. In East Asian, HP-induced immune hyperactivation may drive excessive stimulation of both innate and adaptive immune responses [15], thereby increasing the risk of collateral damage to host tissues. However, the present study does not ensure conclusions regarding immune modulation or disease modification. Third, *rQLT-ACE2/GGT1* exhibits functional involvement in oxidoreductase activity and plays a pivotal role in attenuating oxidative stress. For example, HP-derived lipopolysaccharide (LPS) induces reactive oxygen species that compromise gastrointestinal mucosal integrity. While oxidative stress is biologically relevant to gastrointestinal injury, the current enrichment analysis does not directly demonstrate involvement of oxidative stress pathways [16]. Finally, *rQLT-ACE2/GGT1* contributes to the preservation of gastrointestinal structural and functional integrity by promoting cellular repair and tissue remodeling. During the ulcer healing process, cell survival and extracellular matrix reorganization are critical for the restoration of mucosal architecture and physiological function [17]. However, claims related to extracellular matrix remodeling or collagen deposition are not supported by the enrichment results and are therefore not inferred in this study.

Collectively, *rQLT-ACE2/GGT1* shows a genetic association with DU risk in East Asian populations, with enrichment results suggesting immune-related biological context. However, the current evidence supports only a potential association and does not establish causal effects, therapeutic relevance, or mechanistic conclusions.

## 5. Limitations

This study employed GWAS summary statistics of rQLTs derived from individuals of European ancestry as the exposure dataset, while DU was used as the outcome based on independent GWAS datasets from both European (UK) and East Asian (Japanese) populations. This cross-ancestry design was adopted to evaluate the robustness and generalizability of genetically predicted associations across different ancestral backgrounds.

The European DU outcome dataset (GCST90044132) was derived from UK Biobank–based GWAS data; however, it does not overlap with the proteomic GWAS used for the exposure, as the rQLT summary statistics were generated from independent protein quantitative trait analyses and not from the same GWAS participants used for disease outcome estimation. Therefore, sample overlap between exposure and outcome datasets is considered minimal.

In contrast, the East Asian (Japanese) DU dataset (GCST90270928) was obtained from a separate biobank-based population with distinct ancestry and recruitment sources, further reducing the likelihood of sample overlap with the European UK Biobank exposure dataset.

Given the cross-ancestry nature of the analysis, differences in linkage disequilibrium structure, allele frequencies, and environmental background may contribute to heterogeneity in effect estimates between UK and Japanese. Therefore, the East Asian results should be interpreted as external validation and exploratory evidence of biological consistency rather than direct replication.

Overall, this cross-ancestry MR framework acknowledges the potential heterogeneity among different populations.

## Figures and Tables

**Table 1:** Causal analysis of rQLTs associated with DU.

Forward MR							FDR-Corrected	Reverse MR	Colocalization
Exposure	Outcome	SNPs	OR (95% CI)	*P*_IVW_	*P*_Heterogeneity_	*P*_Pleiotropy_	Adjusted *P*_IVW_	*P*_IVW_	PP_H4_
*rQLT-ACE2/GGT1*	DU (Japanese)	18	0.754 (0.674–0.843)	<0.00001	0.077	0.216	0.0005	0.592	0.999
*rQLT-KLK8/LY6D*		19	0.408 (0.311–0.536)	<0.00001	0.157	0.543	<0.00001	<0.00001	
*rQLT-LY6D/NECTIN4*		23	2.491 (1.971–3.150)	<0.00001	<0.0000	<0.00001	<0.00001	<0.00001	
*rQLT-LY6D/PLA2G10*		28	2.275 (1.908–2.713)	<0.00001	<0.00001	<0.00001	<0.00001	<0.00001	
*rQLT-SCAMP3/VPS53*		12	0.7683 (0.674–0.875)	<0.00001	0.463	0.526	0.042	0.934	0.0002

**Table 2:** Identification of target genes.

Trait/Gene Set	Hub-Genes
*rQLT-ACE2/GGT1* affects DU (Japanese)	*SURF1*
	*SURF6*
	*HLA-F*
	*HLA-A*
	*MED22*
	*HLA-C*
	*SURF2*
	*HLA-G*
	*BCL7B*
	*TBL2*
	*MLXIPL*

## Data Availability

The GWAS summary data used in this study were openly available in the GWAS Catalog, a publicly available, manually curated resource of all published GWAS and association results. The GWAS Catalog is collaboratively produced and maintained by the National Human Genome Research Institute (NHGRI) and the European Bioinformatics Institute (EMBL-EBI). The catalog is accessible at https://www.ebi.ac.uk/gwas/ (accessed on 30 December 2025). The raw data supporting the conclusions of this article will be made available by the corresponding authors on request.
